# Heartworms, macrocyclic lactones, and the specter of resistance to prevention in the United States

**DOI:** 10.1186/1756-3305-5-138

**Published:** 2012-07-09

**Authors:** Dwight D Bowman

**Affiliations:** 1 Department of Microbiology and Immunology, College of Veterinary Medicine, Cornell University, Ithaca, NY, 14853, USA

**Keywords:** Dirofilaria immitis, Heartworm, Drug resistance, Macrocyclic lactones, Ivermectin, Selamectin, Milbemycin oxime, Moxidectin

## Abstract

In order to provide a background to current concerns relative to the possible resistance of heartworms to macrocyclic lactones, this review summarizes various studies in which lack of efficacies (LOEs) have been observed in dogs on macrocyclic lactone preventives relative to the United States of America. Some of these studies have been published in the peer-reviewed literature, others have appeared in various reports to the Center for Veterinary Medicine (CVM) of the Food and Drug Administration (FDA) of the USA as New Animal Drug Application (NADA) summaries, and one appeared as a letter to US veterinarians. This review also discusses reports relating to the potential problem of heartworm resistance in microfilariae and third-stage larvae, as well as molecular markers associated with resistance to macrocyclic lactones within *Dirofilaria immitis*. As more work is being done in this area of great concern relative to the protection of dogs from infection using this class of preventives, it seems timely to summarize what is known about heartworms, their potential resistance to treatment, and the means of selecting for resistance genes in populations of this helminth in the laboratory and in the field.

## Review

Emerging knowledge has brought to the forefront the real possibility that resistance to macrocyclic lactones in the canine heartworm *Dirofilaria immitis* is being observed and could become a threat in the not too distant future. This information appears to contradict what has been observed (or more specifically, not observed) in human medicine, where more than two decades of use of these compounds against similar human parasites have not, thus far, resulted in significant problems of resistance. As study progresses on the apparent loss of efficacy of macrocyclic lactones in canine heartworm prevention, an understanding of the background (e.g., details of the preventive products and their use, issues of compliance, genetics, and other considerations) is necessary to ascertain the presence of true resistance in the heartworm population.

### Heartworm prevention in dogs with macrocyclic lactones and the choice of target doses for preventive treatment

The goal of heartworm preventive therapy in dogs has been to stop infection by *Dirofilaria immitis* by killing the stage that is deposited by the mosquito and first enters the dog, the third-stage larva (L3), as well as the young and maturing fourth-stage larva (L4). The selection of product doses to achieve this goal has often been focused on a minimum effective dose, determined by dose-titration studies using experimentally infected dogs; the dose-limiting target organism, however, has not always been heartworms.

Two types of studies have been established as the routine method for testing monthly heartworm preventives: dose-titration studies to determine the minimum effective dose against the helminth in question, *D. immitis*, and dose-confirmation studies to verify that this minimum dose is effective against two different isolates of the helminth. The testing of the compounds is similar in all cases as per requirements of the Center for Veterinary Medicine (CVM) of the United States Food and Drug Administration (FDA). Thus, the basic study design is the same in almost all cases (with one exception that will be discussed below). Efficacy is determined by giving dogs 30 to 100 L3 of *D. immitis*, and then 30 days later (since the drug is labeled to be given monthly and most products provide very little in the way of residual compound within the dog after just a few days), the dogs in the treatment groups are given the test compound whereas control dogs either remain untreated or are placebo-treated. Approximately five months after infection, all dogs in both groups are euthanatized and necropsied, and the number of worms present in each of the dogs is counted. This is the basic design for both the dose-titration and dose-confirmation trials. In the dose-titration trials, now being called dose-determination trials by the CVM/FDA, there are typically four groups of dogs: one that is treated at the expected target dose, one that is lower than the expected target dose, one that is higher than the expected target dose, and an untreated control group. In the dose-confirmation trials, there are only two groups of dogs – dogs treated with the target dose and untreated controls. Dose-confirmation trials are usually done as replicate studies in two different laboratories, each using a different isolate of the parasite. Due to the perfect efficacy originally afforded by ivermectin, the efficacy for approval of heartworm preventive drugs was set at 100%. Thus, a single worm in a dose-confirmation trial would preclude a drug from receiving approval. The single exception to this basic experimental design for the testing of all the products on the market in the United States is ProHeart® 6, a slow-release injectable product designed to prevent infection for six months. Thus, for the ProHeart® 6 studies, dogs in the treatment group are administered the product, and then six months later both the treated dogs and the untreated control dogs are inoculated with 30 to 100 L3; five months after infection, these dogs are euthanatized and necropsied to determine worm burdens. The difference in design is because this drug is expected to be present in the dogs for six months, and thus, at six months it must still provide 100% protection against newly acquired infections. To reiterate, with most preventive products the drug is cleared fairly rapidly, and the dogs are dosed monthly to kill any larvae that have been acquired during the last 30 days. In contrast, the level of ProHeart® 6 remaining within the dogs six months after treatment is intended to be sufficient to provide 100% protection against incoming larvae. These are the two basic formulas for all the trials presented below.

For Heartgard 30® (As marketed: tablets formulated to supply a minimum effective dose band of 6 mcg ivermectin/kg and a maximum dose of 12 mcg/kg), the target was heartworm prevention. In the original NADA 138–412 [1], "Of the 83 dogs treated at monthly intervals in natural infection trials, or treated 30 days after induced infection, with doses of ivermectin at 3.0 mcg/kg or greater, only 2 dogs developed infections [Author’s note: the 2 dogs that were positive were dogs that had each received 3.0 mcg/kg on day 30 after having been infected with approximately 50 L3]. Even when the treatment interval was extended to 45 or 60 days following infection, only 2 of 88 dogs given ivermectin at 6.0 mcg/kg or more developed infections." Dogs receiving 6 mcg/kg at 45 and 60 days were negative at necropsy. Thus, the minimal effective dose of 6.0 mcg/kg monthly was chosen.

In the case of Interceptor® (As marketed: formulated in a tablet to provide a dose band of 0.5-1.0 mg milbemycin oxime/kg) (NADA 140–915), 0.05 mg/kg was not always 100% protective against experimental heartworm infections when administered 30 days post inoculation with L3 [2]. However, a single treatment with 0.1 mg/kg or more at 30 days post infection appeared 100% effective [2]. The higher dose of 0.5 mg/kg was eventually chosen for this product in order to achieve >90% efficacy against hookworms.

In the case of Revolution™ (As marketed: formulated as a topically applied product to deliver a dose band of 6–13 mg selamectin/kg) (NADA 141–152) [3], the dose was driven by flea control where "6 mg/kg of selamectin was selected as a minimum dose for effectiveness against fleas on dogs 30 days following a single topical administration." For heartworms, "Selamectin applied topically as a single dose of 3 or 6 mg/kg was 100% effective in preventing the maturation of heartworms in dogs following inoculation with infective *D. immitis* larvae 30 or 45 days prior to treatment, and 6 mg/kg was 100% effective in preventing maturation of heartworms following inoculation of infective larvae 60 days prior to treatment."

In the case of ProHeart™ Tablets (As marketed: tablets formulated to provide a dose band of 3–6 mcg moxidectin/kg) (NADA 141–051) [4], "Moxidectin was 100% effective in preventing the development of a one month-old heartworm infection of 50 L3 larvae of *D. immitis* in dogs when administered as a single oral treatment at 1.5, 3.0, and 6.0 mcg/kg." In this NADA there were no dose-confirmation studies with dogs receiving the 3.0 mcg/kg target dose a month after experimental infection with 30 to 100 heartworm larvae. However, there was a second dose-determination trial where “Moxidectin was 100% effective in preventing the development of a two month-old heartworm infection of 50 L3 larvae of *D. immitis* in dogs when administered as a single oral treatment at 0.5 mcg/kg.” The target dose chosen for ProHeart™ Tablets was 3 mcg/kg.

For ProHeart® 6 injectable (As marketed: formulated as an injectable slow-release product providing an initial dose of 0.17 mg moxidectin/kg; there is no dose band due to the formula allowing precise dosing) (NADA 141–189) [5], dosage titrations were performed with initial doses of 0.06, 0.17, and 0.50 mg/kg. In one study no heartworms were recovered from any dogs receiving any of the moxidectin-containing products, while in the other study one dog in the lowest dose group was infected. Thus, the minimum dose of 0.17 mg/kg was chosen. This dose was also found to have excellent efficacy against hookworm infections.

For Advantage Multi® for dogs (As marketed: formulated in a topical application to provide a combination product with a dose band of 2.5-6 mg moxidectin/kg for heartworms and 10–25 mg imidacloprid/kg for flea control) (NADA 141–251) [6], the dosage was chosen based on intestinal nematodes - "Dosage Characterization for the Prevention of Heartworm Disease: Refer to section e., Dosage Characterization for the Treatment and Control of Intestinal Nematodes, which establishes a minimum effective dose for moxidectin." There was not a dose titration performed since the target parasite was not heartworm. In three trials, however, this minimum dose of 2.5 mg moxidectin/kg protected all 44 dogs that were treated 33 to 34 days after inoculation and 12 dogs treated 45 days after inoculation.

### Control of filariid infections in humans

In human medicine, the target dose for the control of the human filariid nematodes *Onchocerca volvulusWuchereria bancrofti*, and *Brugia malayi* has not been chosen for the purpose of preventing infection by killing L3 and L4. Rather, the goal has been to suppress the levels of microfilariae in the blood and skin of infected individuals so that, although the adult worms survive, there is no transmission between people. Also, the suppression of the skin-dwelling microfilariae of *O. volvulus* prevents the damaging effects of the microfilariae on the cornea - the cause of river blindness. The dose chosen for microfilarial suppression has been 200 mg/kg administered orally every 6–12 months [7]. This increased dosage is necessary because microfilariae are typically less susceptible to macrocyclic lactones than are immature (L3, L4) filariid larvae. Although the campaign against human filariasis has now been active for more than twenty years with millions of doses being given to people in the developing world, resistance has not emerged as a significant problem; however, there are some very recent indications that the duration of microfilarial suppression may not be as long as it once was after treatment [8]. As yet, these effects do not appear to be hampering the progress of control in the field.

### Factors that may influence the development of resistance in heartworms

Two factors in the prevention of heartworm infection are both likely to play major roles in how the different drugs might lead to resistance or how they might differ in their ability to affect resistant worms. First, as discussed above, canine heartworm preventives were designed to prevent infection by killing L3 and young L4. They were not designed to suppress microfilariae as in the control of human filarial infections. If drugs at preventive doses are given to dogs with existing infections, microfilariae will not be suppressed and many will survive in spite of the fact that they have encountered a macrocyclic lactone; these drug-selected microfilariae can then be transmitted between dogs by mosquitoes. Thus, use of preventives in microfilaremic dogs is a less than prudent course since it might select for resistance through the exposure of microfilariae to sublethal concentrations of macrocyclic lactones. Secondly, some of the heartworm preventives on the market had other targets, such as fleas and intestinal nematodes, that required higher doses for killing than did heartworm, resulting in an increased minimum dosage for the combination product. Very typically in the case of resistance, increased doses of drug will continue to be efficacious against resistant strains until selection is so marked that the treatments become toxic to the host before the worms are killed. In either case, with a resistant phenotype, if dogs are infected by L3 that develop to patency in spite of exposure to a preventive, the ever-hardier microfilariae will be capable of transmission by mosquitoes to other dogs. The concern is whether or not resistant phenotypes of heartworms have already appeared in the field.

### What is resistance?

Resistance is defined by there being "a greater frequency of individuals within a population able to tolerate doses of a compound than in a normal population of the same species and is heritable……” [9]. Resistance to macrocyclic lactones is a well known phenomenon amongst intestinal nematodes. It has appeared in *Haemonchus contortus* populations [10]; in populations of *Parascaris equorum*[11]; and recent concerns have arisen relative to the small strongyles of horses [12]. In these cases, the target for control has been the adult worms using formulations with 90% to 98% efficacy. Resistance is selected by the repeated treatment of all animals, placing significant selection pressure on the populations within the intestine of the host. The worms that survive treatment exposure are the only worms producing eggs for a period of time, and this provides them with a competitive edge. In the case of *Haemonchus contortus*, resistant isolates have been maintained in sheep, and the heritable resistance trait is known to pass from generation to generation [13].

Potential arguments against the development of resistance within heartworm populations might be the long generation time, i.e., the 7-month-long life cycle, large numbers of untreated refugia (stray dogs and, in the USA, coyotes), and the perfect efficacy of the preventive products against the highly susceptible L3. However, the long life cycle and refugia are unlikely to play a role if, for the former, resistant worms do not make a significant trade-off in return for fitness, and for the latter, because full reversion (i.e., loss of resistance) - as sometimes occurs in bacteria when the drug pressure is removed - has not been observed in nematodes [14]. Also, the killing of a highly susceptible L3 stage by low doses of product may not be of actual significance in delaying resistance if, in the field, another more numerous and more resistant stage, such as the microfilaria, becomes the target of misdirected off-label treatment. That resistance persists through worm developmental stages is indicated by the success of the larval migration assay for detecting resistance of adult *H. contortus* to macrocyclic lactones [15].

Since drug resistance in nematodes does not seem to impair fitness, the detection of this trait in any nematode population is of significant concern. Treatment failures in the field may be due to resistance, but often the true cause is hard to pinpoint due to potential problems with recording, lack of compliance, underdosing, reinfection (or preinfection in the case of heartworm preventives), etc. For some compounds, it is possible that resistance is not inevitable for a plethora of factors that may be genetic or related to management. As resistance of nematodes to various compounds, including macrocyclic lactones, has been reported in a number of important nematode parasites, it would seem that macrocyclic lactone resistance amongst filariid nematodes is a real threat. The current debate is whether or not the recent reports of lack of efficacies (LOEs) with heartworm preventives are due to the appearance of resistant forms or a confluence of confounding factors such as poor compliance.

### The Mississippi delta and lack of efficacies (loes)

In the area extending along the Mississippi River from Tennessee through Louisiana, there are many practitioners who contend that the heartworm preventives they have been using are no longer protecting dogs from infection. On the other hand, it has been reported that a very high percentage of these product failures are occurring in pets where compliance has not been as good as initially perceived by the claimant [16]. This speaks to the continuing problem of trying to identify resistance in the field, where treatment failures may or may not accurately reflect an underlying issue of resistance. However, other knowledge has come to light in the past 18 months that suggests that the veterinary community should be concerned about the threat of heartworm resistance.

### Microfilarial studies related to resistance phenotypes

Microfilariae have been purified from the blood of dogs in the Mississippi Delta that were purportedly infected with heartworms while on preventive therapy. These microfilariae were examined for their ability to survive in different concentrations of several macrocyclic lactones [17]. Some of the isolates showing reduced susceptibility to macrocyclic lactones were grown to the L3 stage in mosquitoes and used to infect dogs. When the microfilariae from these latter dogs were examined in the same assay, they were found to similarly lack susceptibility to macrocyclic lactones. Thus, this observed lack of microfilarial susceptibility to macrocyclic lactones is a form of genetically inherited resistance; however, this trait may or may not be linked to the ability of later stages (i.e., L3 and young L4) to grow to adulthood in dogs on preventive therapy. With the isolation of these strains, experiments can now be designed to test whether these isolates can successfully infect dogs on preventive therapy.

### Persistent microfilaremia in a Katrina rescue dog taken to Canada and treated for its heartworm infection

Anecdotal reports by practitioners have circulated about the inability to clear dogs of their microfilariae after the adults are removed with Immiticide® (melarsomine dihydrochloride). Recently, a detailed case description was published concerning one such case [18]. This was a dog from the southern United States that was rescued following hurricane Katrina and relocated to Canada where it was treated with Immiticide® for its heartworm infection on two separate occasions, 5 months apart [18]. The dog remained microfilaremic after the first treatment in spite of being placed on a macrocyclic lactone preventive product. Eight months after the first adulticidal treatment, the dog had become antigen negative, but it remained positive for microfilariae in spite of having received a second round of Immiticide® and multiple treatments with macrocyclic lactones including two doses of ivermectin at 200 mcg/kg. The dog was then treated every other week with milbemycin oxime, ultimately near the top of the preventive dose band at 1.1 mg/kg, and finally daily for 7 days at 2.0 mg/kg followed again a month later by the daily administration of 2.0 mg/kg for 8 days. The dog remained antigen negative and microfilariae positive until just recently when the microfilariae finally disappeared, more than two years after the second adulticidal treatment (Personal communication with one of the authors). This appears to be independent verification that microfilariae that are refractory to macrocyclic lactones exist [17]. However, microfilariae from this infection were not grown to L3 and used to infect another dog, so it is not known whether this resistance trait was inheritable, and again, there is no proof that the phenotype of microfilarial resistance translates into resistance of L3 when they are inoculated into dogs receiving macrocyclic lactones.

### Susceptibility of third-stage larvae from mosquitoes to macrocyclic lactones

Infective-stage heartworm larvae have been examined for resistance to macrocyclic lactones using an assay where L3 grown in mosquitoes are allowed to migrate through fine mesh in the presence of different macrocyclic lactone concentrations (a method originally developed to measure the prevalence of resistant phenotypes in horse and sheep strongylids). L3 grown from the apparently resistant microfilarial isolates from the Mississippi Delta were found to be as susceptible to macrocyclic lactones as L3 grown from non-resistant microfilariae [19]. However, as with microfilarial susceptibility, it remains unclear whether the phenotype of decreased L3 susceptibility to macrocyclic lactones is related to the ability to infect a dog on heartworm preventive therapy.

### Lab-based studies with heartworms in dogs relative to preventive product failures

In 2004, Pfizer Animal Health sent a letter to all veterinary practitioners in the United States that informed them that "An additional experimental study evaluating the performance of Revolution® involved mixed breed dogs challenged with a rigorous infection of 50 heartworm larvae and treated 30 days later with a single dose of Revolution™. …. The results indicated some treated dogs harbored 1 or 2 adult heartworms five months following this laboratory challenge, all untreated control dogs exhibited substantial worm burdens (14–43 adult worms per dog)." This was the first report of laboratory studies in which heartworms developed in dogs given a single dose of a marketed preventive at the prescribed dosage 30 days after inoculation with infective larvae of a laboratory strain.

#### The development of Trifexis®

In 2011, two papers and the Freedom of Information summary for the New Animal Drug Application (NADA) appeared on the development of a new milbemycin oxime-containing heartworm (HW) preventive product, Trifexis® (As marketed: tablets formulated to provide a dose band of 0.5-1.0 mg milbemycin oxime/kg ion for heartworm with 30–60 mg spinosad/kg for flea control) [20-22]. "To achieve a FDA-CVM-approved label claim for HW prevention as determined in dose confirmation (DC) testing protocols for these pioneer ML [macrocyclic lactones], dogs were generally inoculated with 50 infective *D. immitis* third stage larvae (L3) obtained from experimentally infected mosquitoes, and then 30 days later administered a single dose of the preventive being tested. A corresponding nontreated control group was used to confirm adequacy of infection of the HW isolate. Five to 6 months later, after surviving worms have had a chance to mature, necropsy examinations determine the effectiveness of the product. The presence of a single HW in any of the treated dogs would have prevented approval because 100% prevention was necessary to obtain a label claim based on this dosing protocol. ….. As part of the development program of an ML for HW prevention, 2 separate recently isolated HW isolates were tested according to current FDA-CVM requirements. While one isolate was fully susceptible to this ML [macrocyclic lactone]providing 100% prevention after a single dose administered 30 days after inoculation with HW L3, efficacy of a single treatment against the second isolate was <100% [20]." In this study, 3 of the 10 infected dogs treated with Trifexis® had 3 worms at necropsy, 1 had 1 worm and 2 had 2 worms. This work was done with a strain identified as MP3 - a heartworm isolate named after the naturally infected dog from Georgia, USA from which it was isolated, “Miss Piggy.”

The manufacturer of Trifexis® pressed forward with its development and reported "A study was undertaken with this second field isolate to assess the effectiveness of currently marketed ML, in this case administering a single dose of IVM [ivermectin] or MBO [milbemycin oxime], in dogs challenged with HW L3 1 month before treatment [21]." The products examined for preventive efficacy were Heartgard Plus Chewables for Dogs® (As marketed: dose band of 6–12 mcg ivermectin/kg), and Interceptor Flavor Tabs for Dogs & Cats® (As marketed: dose band of 0.5-1.0 mg milbemycin oxime/kg). This work was done in dogs with the MP3 strain using a similar challenge model as already described [20]. There was one worm found in one dog in each of the Heartgard Plus®- and Interceptor®-treated groups of dogs (comprising 14 dogs each). Thus, neither product was 100% efficacious in preventing infection by the MP3 strain in this historical method of preventive testing.

In the development of Trifexis®, additional studies were conducted with this strain examining the effects of multiple treatments at 30 and 60 days after infection and at 30, 60, and 90 days after infection. In the 10 dogs treated twice with Trifexis®, there was a single worm present at necropsy at 150 days postinfection (1 worm in 1 dog), whereas no worms were observed in the dogs treated three times. Thus, the Trifexis® label has the statement "For heartworm prevention, give once monthly for at least 3 months after exposure to mosquitoes “[22]. Continuing treatment for three months following the end of the mosquito season should protect dogs from infection by killing any worms acquired during the last month of transmission.

#### MP3 and Advantage Multi® versus Heartgard Plus®, Interceptor Flavor Tabs®, and Revolution™

In the introduction to a study that compared the efficacy of these four products in dogs with a single treatment 30 days after inoculation with 100 L3 of the MP3 heartworm isolate, it is disclosed that the sponsor of the research, Bayer Animal Health, LLC, as part of product development, was working on a new macrocyclic lactone-containing preventive (two formulations of ivermectin-containing products with target minimum doses of 6 mcg/kg and 9 mcg/kg; personal communication as to dose bands used) [23]. In this work using the same dose of ivermectin as an already marketed product or one that was at a dose 1.5 times higher, "a strain of *D. immitis* (MP3 laboratory strain; TRS Laboratories Inc., Athens, GA) was used to evaluate a potential new anthelmintic product." It was found "In that study, the MP3 laboratory strain was less susceptible to traditionally effective doses of an ivermectin-based preventive in a limited number of dogs." Thus, Bayer decided to examine the efficacy of four commercial monthly products against this problematic strain.

The study design in this subsequent trial used 100 L3, treatment with the labeled monthly dose 30 days later, and necropsies 150 days after treatment. There were 8 dogs in each group. The results were such that 7 dogs developed infections with adult worms in each of the groups treated with Heartgard Plus® (0–7 worms), Interceptor® (0 to 6 worms), and Revolution™ (0–9 worms), whereas no worms were recovered from any 8 of the Advantage Multi®-treated dogs. Thus, again, with the MP3 isolate, not all dogs were afforded 100% protection by all products. In these single treatment studies, only Advantage Multi® was 100% efficacious in preventing development of the MP3 larvae to adulthood.

#### Summary of the above studies utilizing MP3

Among the studies discussed above, where the MP3 strain was used, there have been eight reported instances in which dogs have become infected in spite of being given a single heartworm preventive treatment 30 days after infection: 1 study with Trifexis®, 2 studies with Heartgard Plus®, 2 studies with Interceptor®, 1 study with Revolution™, and 2 studies with ivermectin in a product undergoing development. There was also one study where one dog had one adult heartworm at necropsy after having received two Trifexis® treatments 30 and 60 days after infection. Although F1 generations of MP3 have been established (personal communications to the author), there have been no reports as of yet on whether the resistance trait seen with MP3 is heritable. One might surmise, however, that worms that survive a single treatment are perhaps more likely to produce offspring with a greater chance of surviving repeated macrocyclic lactone preventive therapy than those killed by a single treatment. Of course, it depends on whether the trait is present in the genome of the worm and how it is inherited by offspring when the worms mate.

#### Additional studies with lack of efficacy

There has recently been another report of dogs not being protected with milbemycin oxime at the same dosage as provided in Interceptor®, Sentinel®, and Trifexis®. In this case, Sentinel® Spectrum® Tasty Chews (a combination product of 0.5-1.0 mg milbemycin oxime/kg, lufenuron, and praziquantel) was being brought to market in the USA (NADA 141–333) [24]. The report in the NADA approved in December of 2011 includes a trial wherein the dogs were treated monthly for 6 months beginning 30 days after infection with 50 *D. immitis* L3. In this study, all treated dogs were protected. However, the NADA states: "A 6-consecutive monthly dosing regime was selected for effectiveness studies against *D. immitis* infections. Neither one dose nor two consecutive doses of SENTINEL SPECTRUM provided 100% effectiveness against induced heartworm (*D. immitis*) infections in dogs". The label for this product states: "For heartworm prevention, give once monthly beginning within 1 month of the dog’s first seasonal exposure to mosquitoes and continuing until at least 6 months after the dog’s last seasonal exposure." At this point, it is unclear if this study was done using the MP3 strain, but the work was performed in the laboratory where the strain was first isolated and maintained, so it is a possibility. If the strain was MP3, it would mean that there is an additional study where milbemycin oxime was not effective in preventing the development of heartworms after one or two treatments.

### Molecular biological examination of heartworms as related to resistance

Recently the molecular phenotypes of heartworms have been examined in an effort to see if various molecular markers may be associated with treatment failures [25,26]. The amount of polymorphism has been examined in different genes associated with macrocyclic lactone resistance in other nematodes, e.g., the ABC transporter gene - best known to veterinarians as the PGP (P-glycoprotein-like) gene or the MDR1 (Multi-Drug Resistance 1) gene - that is defective in dogs of the collie breed, leading to avermectin sensitivity. It was first shown that in heartworms collected from around the world, there were small differences in several of these genes that appeared to be randomly distributed throughout the population, i.e., the worms were polymorphic within the gene examined. [Author’s note: A gene exhibits no polymorphism if any change in the gene is fatal to the worm, and if there is no polymorphism in a gene, then it is of no value in determining whether or not drug selection has an effect on the frequency of polymorphism. Selection will produce reduced polymorphism in a gene within a population; this is the classic selection for pea color that led Mendel to his understanding of genetic heritability.] In the case of *D. immitis*, these authors found that the microfilariae produced by isolates from the Mississippi Delta were markedly reduced in their polymorphism for the genes examined. The authors concluded that this indicated that selection, likely via macrocyclic lactones, had driven worms toward genetic similarity in those genes. Any selection in nematodes is believed to be housed solely in the genome, so any reduced polymorphism would also appear in all life stages. These same investigators found a reduced polymorphism in the microfilariae from the Katrina rescue dog in Canada whose microfilariae did not clear in the presence of high doses of milbemycin oxime [18]. However, they have also examined these genes in MP3 and have found that, based on the polymorphisms examined, the genes from the MP3 strain appear susceptible but with some degree of ML selection in its history but that they have not undergone a high degree of loss of polymorphism when compared to that of the Mississippi Delta isolates and the Katrina rescue dog [27].

### So – do we have resistance?

Resistance can come about in several ways. It can be due to a spontaneous mutation. It can be induced by mutagens, e.g., irradiation or chemical exposure. It can be due to continued selection pressure - such as by repeated treatment of worms with the same drug – causing a particular phenotype to rise in frequency within a population. In the case of heartworms, we can probably rule out a mutagen-induced introduction of resistance genes; thus, it is most likely that the resistance is due to either a spontaneous mutation or via selection of a rare or uncommon phenotype.

#### Mp3 as a spontaneous mutation

If with MP3 the resistance is heritable, i.e., microfilariae produced by dogs infected with L3 derived from Miss Piggy’s microfilariae are capable of infecting dogs receiving macrocyclic lactone preventives, then this is a strain that is resistant to macrocyclic lactones. (It can be argued that it is not fully resistant because multiple preventive doses are protective, but it can also be readily argued that it is a phenotype that undoubtedly has a reduced susceptibility to macrocyclic lactones compared to other strains that have been historically examined.) It does not matter whether Miss Piggy’s population of adult worms and their microfilariae ever saw macrocyclic lactones before or not. If these microfilariae – which have been shown to be less susceptible to heartworm preventives – grow to adulthood and produce offspring that can routinely infect dogs on preventives just like their parents, they are resistant worms. This could be due to a one-time chance event occurring from the pairing of one male and one female worm within Miss Piggy producing microfilariae with a resistant phenotype. It may be that MP3 was simply a rare capture of a spontaneous mutation.

### MP3 chosen by Selective Drug Pressure

It is also possible that the genes for resistance have been in the genetic make-up of a small population of *D. immitis* somewhere in the field for a very long time, and via the widespread use of macrocyclic lactones, worms in dogs were selected that have a resistant phenotype. These already resistant worms then found their way via a mosquito into Miss Piggy. Since both the ancestors of filarial nematodes and the Actinobacteria producing the macrocyclic lactones occur within soil ecosystems, genes supporting resistance to these bacterial products have likely provided a survival advantage to nematodes for eons. The genes only became important relative to drug resistance in nematodes like *Parascaris equorum* and *H. contortus* when we started using purified products from these Actinobacteria to treat or prevent nematode infections. Then, these resistant phenotypes were promoted by selection. In this scenario, the genes for resistance were already present in the population and just never had a chance to show their survival potential until challenged, such as occurred when the MP3 isolate was captured and tested in the studies described above.

When parasitologists think and talk about resistance to anthelmintics they are usually considering the drug-induced selection of resistant forms. The selection of MP3 and possibly other heartworm strains in the United States with resistance to macrocyclic lactones could occur in at least two different ways. In one case, where selection pressure may have been applied through regular preventive therapy, worms like MP3 - representing a very small portion of the population - might sneak through and survive in a few dogs on preventive. These infections might remain undetected if dogs with developed adults and microfilariae did not receive annual status checks, and the patent infections could then be spread by mosquito transmission. In another case, the adulticidal treatment of dogs with macrocyclic lactones (Slow-Kill or Soft-Kill) rather than Immiticide® might, by subjecting the off-target microfilariae to prolonged drug exposure, select for populations of resistant circulating microfilariae that are spread to new dogs by mosquitoes. In the first case, selection is at the level of L3 and L4; in the second case, selection would be occurring at the level of the microfilariae.

## Conclusions

At this point, it is not known if the F1 generation of worms produced from microfilariae of LOE dogs in the Mississippi Delta can develop to adults in dogs on preventive therapy. We do not even know at this time if they can survive following a single preventive treatment, as has MP3 in clinical drug trials. Based on the data to date, it appears that the microfilarial assay used to evaluate these isolates may measure a lack of susceptibility in microfilariae, but this may not correlate with increased survival of L3 (grown from those same microfilariae) in the presence of these anthelmintics. Also, the isolates from the Mississippi Delta and the Katrina dog that have reduced sensitivity to macrocyclic lactones were different than MP3 in that MP3 microfilariae appeared to be more similar to the microfilariae of fully susceptible forms based on certain gene polymorphisms. It is possible that the phenotype being examined in the microfilarial susceptibility assay might not be the same as that required for resistance in larval and adult heartworms.

If the offspring of MP3 are found to behave the same way as the original isolate in similar drug efficacy trials, the trait is heritable, and resistance could be assumed. It does not matter if the trait was due to spontaneous mutation or selection. Again, because heritability of this trait has yet to be shown for MP3, it cannot be stated whether the resistant phenotype will persist into the next generation. A spontaneous inheritable mutation event would seem likely to occur with less frequency than cases selected for by large-scale drug use in a population where some worms already carry resistance genes. If the problem with selamectin as reported by Pfizer’s 2004 letter was due to a difference in the isolate used in those trials, rather than due to some defect in product absorption or other product-associated problem, then this could be considered evidence of either spontaneous mutations occurring on more than one occasion or the existence of a survival-promoting genetic trait in a number of heartworms within the general heartworm population. Again, from the original Heartgard® 30 NADA 138–412, there were two dogs that developed heartworm infections out of 83 dogs treated at 3.3. mcg/kg administered 30 days after infection, and 2 dogs given 12 mcg/kg ivermectin developed adult heartworm infections with 1 and 5 worms each, when product was administered at 45 days and 60 days after infection (dogs getting 6 mcg/kg at 45 and 60 days were negative at necropsy). These results from sometime before 1987 suggest that there is polymorphism in the worms relative to a potentially resistant phenotype. This would lead to the conclusion that there are resistance-associated phenotypes occurring at some low level in the *D. immitis* population.

Recently, there has been another study of the genetics of heartworms in the United States. Using 11 polymorphic microsatellite loci and 192 individual heartworms from 9 different geographic locations in the United States and Mexico, the genetics and population structure of heartworms were examined and 4 major genetic clusters were identified [28]. The clusters were associated with the eastern United States, central USA, western USA, and Mexico. There was a low level of heterozygosity observed in general, and high levels of reciprocal gene flow between the populations in the eastern US and the central US. It appears that geographic barriers impede significant gene flow between these areas and California and Mexico. Germane to the conclusion of this review is a conclusion reached by these authors on the relationship between gene flow and resistance: “This pattern of gene flow could certainly influence the spread of alleles beneficial to canine heartworm. In an area where there is a significant amount of gene flow, such as the Gulf Coast, the dispersal of drug resistance alleles would occur rapidly. Those resistance alleles would not necessarily need to arise in that geographic region, but could arrive there via dispersal from some other area.”

At this point, careful study has begun into the issue of resistance in heartworms, and the potential of resistance existing has been taken seriously in many different laboratories. The life cycle of heartworms is long, and it takes at least 6 months to develop adult worms and produce circulating microfilariae in a dog, and then another 6 months to determine if these microfilariae, when grown to L3 in mosquitoes and used to infect additional dogs, produce resistant worms. It also must be remembered that worms, unlike bacteria, have to undergo genetic recombination through mating between males and females to produce offspring. What if there is only one adult male in the MP3 population containing the resistance trait? There is a good chance that a similar male may not develop in the group of 30–50 worms in the next infected dog. At this time, we do not even know that the worms from an MP3 infection are capable of producing microfilariae if the infections develop and persist in the face of preventive therapy; the assumption is that they would, but they might not. Overall, a prudent approach remains similar to what has been recommended in the past: Vigilance through testing dogs before initiating heartworm prevention; vigilance through testing dogs annually for heartworms to avoid letting any MP3 (or other potentially resistant strain) slip by, live, and produce microfilariae while the dog is on prevention; and vigilance through treating dogs with adult worms with Immiticide® rather than a macrocyclic lactone, to reduce the chance of producing or selecting microfilariae with a resistant phenotype. Furthermore, it would be prudent to keep all dogs on year-round prevention since this should reduce selection of worms that have seen only one dose of drug due to a miscalculation of the end of the transmission season, or because it would appear that some strains, such as MP3, may require three consecutive doses of preventive to be fully eliminated. It is critical that dogs on preventive be tested annually and, if found infected, that the infection be cleared using Immiticide®. There is some indication that doxycycline administration to dogs with adult worm infections might suppress the infectivity to the next canine host of L3 that develop from microfilariae produced by these doxycycline-treated worms [29], but the sample size from this work is still very small, so it may not be wise to count on this quite yet. Overall, at this time, there is reason for concern, a lot of research that still needs to be performed, a critical need for veterinarians to impress upon their clients that they need to pay attention to keeping current on their prevention, and a need for veterinary practitioners to practice careful record keeping while continuing to report all LOEs to the FDA.

## Competing interests

The author has, in the past, received financial remuneration from the manufacturers of all products mentioned in this review.
